# Narrative recognition and identification: a qualitative pilot study into reading literary texts with advanced cancer patients

**DOI:** 10.1007/s11764-021-01048-0

**Published:** 2021-06-15

**Authors:** Albert Kamp, Zarah Bood, Michael Scherer-Rath, Yvonne Weeseman, Nirav Christophe, Henny Dörr, José Sanders, Mirjam Sprangers, Esther Helmich, Liesbeth Timmermans, Ellen van Wolde, Hanneke W. M. van Laarhoven

**Affiliations:** 1 Faculty of Philosophy, Theology and Religious Studies, Radboud University, Nijmegen, The Netherlands; 2 Department of Medical Oncology, Cancer Center Amsterdam, Amsterdam University Medical Centers, University of Amsterdam, Meibergdreef 9, Amsterdam, The Netherlands; 3 HKU University of the Arts Utrecht, Utrecht, The Netherlands; 4 Centre for Language Studies, Radboud University, Nijmegen, The Netherlands; 5 Department of Medical Psychology, Amsterdam Public Health, Amsterdam University Medical Centers, University of Amsterdam, Amsterdam, The Netherlands; 6 Amsta Healthcare Organisation, Amsterdam, The Netherlands; 7 Department of Primary and Community Care, Radboud University Medical Centre, Nijmegen, The Netherlands

**Keywords:** Narrative identification, Reading guide, Experiences of contingency, Life story, Patient care, Quality of life

## Abstract

**Purpose:**

Patients with advanced cancer can experience their disease as a contingent life event. The sudden interruption of their life stories can obscure life goals and disrupt meaning making. In the context of the research project “In search of stories,” we aim to investigate the reading and discussion of selected stories which present ways of dealing with a contingent life event. In addition, we examine the use of a newly developed guide for reading these exemplary texts together with advanced cancer patients.

**Methods:**

This qualitative study describes the experiences of five patients with advanced cancer who participated in a guided reading and discussion about selected literary texts. The intervention consisted of reading a selected story, after which each patient was interviewed, using the reading guide as a conversation template. The interviews were then thematically analyzed for their conceptual content using a template analysis.

**Results:**

All five conversations showed some form of recognition in reaction to the chosen text, which led to personal identification of experiences of contingency, such as loss of life goals, impending death, or feelings of uncertainty. Besides the important role of identification, revealed by the responses to the questions in the reading guide, the discussion of the text helped them articulate their own experience and sources of meaning. Diverse worldviews came to the fore and concepts of meaning such as fate, life goals, quality of life, and death.

**Conclusions:**

First experiences with our newly developed reading guide designed to support a structured reading of stories containing experiences of contingency suggest that it may help patients to express their own experiences of contingency and to reflect on these experiences.

**Implications for Cancer Survivors:**

The intervention tested in this study may contribute to supportive care for survivors with advanced cancer, but further research is needed to evaluate its effect on quality of life.

## Introduction

Falling seriously ill, for example when people are diagnosed with incurable cancer, has a profound impact on people’s personal lives and their individual life stories [1]. The illness not only affects a patient’s physical health but his/her psychological and spiritual wellbeing is also at stake [2]. Being diagnosed with a life-threatening disease places pressure on a patient’s life expectations and can be experienced as a contingent life event. Contingency refers to the idea that everything could have been different, and compared to one’s plans and expectations could develop otherwise [3]. The occurrence of the life event is a possibility, not a necessity. This experience of contingency can be described as a crisis of meaning and interpretation, something that complicates the progress of the patient’s meaningful life story [4]. In cases where the cancer diagnosis leads to an unexpected disruption of a patient’s life, the patient has to reinterpret his/her life story.

The process of reinterpretation, to search for and express meaning in life, relates to the concept of spirituality [5]. According to Puchalski et al. (2009, p. 887), “Spirituality is the aspect of humanity that refers to the way individuals seek and express meaning and purpose and the way they experience their connectedness to the moment, to self, to others, to nature, and to the significant or sacred.” [6]. Several studies have shown the importance of spiritual care in advanced cancer patients [7–9]. Despite an increasing recognition of the pertinence of spirituality in dealing with a terminal illness [10], it is still underappreciated in the palliative care of oncological patients, and patients have reported unmet spiritual needs [11].

In randomized clinical trials, spiritual interventions addressing existential themes using a narrative approach have shown positive effects [12]. However, effect sizes were moderate, which may, among others, be due to the common understanding that attribution of meaning to experiences of illness is an individual process that people should experience alone. However, patients can find themselves “empty handed” when it comes to creating meaning or constructing a meaningful life story [13, 14].

In order to contribute to the process of meaning making of advanced cancer patients, in the research project “In search of stories,” we set out to develop a narrative intervention. Under the guidance of a spiritual counsellor, patients are provided with a selection of literary texts in which ways of dealing with a contingent life event are presented. The exemplary stories are a starting point for the conversation about a patient’s own life story. After discussing their life story, patients engage in a co-creative art-making project with a professional artist, empowering them to create their own new life story [3, 15].

In this study, we focus on the part of the project in which literary texts are read and discussed. These texts offer patients the possibility to recognize similar experiences and introduce them to other views and interpretations. In this way, the patients could distance themselves from their own perspective and look at their own situation in new ways. Central to this process is the notion of narrative identification. On the one hand, from a cognitive linguistic point of view, some form of identification is necessary in the process of reading literary texts, such as recognizing words and concepts, in order to create coherent mental representations of these concepts [16] and to comprehend the narrative presented as a meaningful story [17]. A cognitive linguistic approach focuses on the cognitive processes by which our interaction with the world is mediated through informational structures in the mind.

In addition to the recognition of situations and events, it is important how patients identify with characters in the story, whose visions on the events are presented [18]. After all, while reading, readers adopt the goals and plans of characters, causing them to experience emotions as the story unfolds [19].

On the other hand, narrative identification points to the psychological process that individuals form an identity by integrating life experiences into their personal life story [20–22]. The connection of life experiences to concepts of meaning and worldview refers to the spiritual component which, in a way, forms a further elaboration of the psychological process of narrative identification.

Offering patients exemplary narratives could stimulate a process of narrative identification in a cognitive linguistic, psychological, and spiritual respect. This process starts by recognizing words and concepts (linguistic), followed by identifying with subjects and situations (psychological), and connecting these to the patient’s own life experiences and worldview (spiritual) [23]. The extent to which this connection takes place depends to a considerable degree on the perceived similarities between the main character and the reader [24].

We report on our first experiences with reading and discussing exemplary literary texts with advanced cancer patients, using a newly developed reading guide to assist the process of narrative identification.

## Methods

### Selection of literary texts

The literary texts were selected by the following authors who all have a different expertise relevant to the project: clinical oncology and palliative care (HvL), medical psychology (MS), qualitative health research (EH, ZB), language studies and narrative discourse (JS), religious studies and theology (MSR, EvW), performative processes in arts (HD, NC), and primary and community care (LT). The selection process consisted of three phases: (1) collection of relevant book and story titles suggested by all authors; (2) selection of specific text fragments from the suggested books by ZB, NC, and HD; (3) discussion and final selection of the passages by ZB, NC, HD, HvL, and MSR.

In steps 2 and 3, the following criteria were used: the story is written in a literary style, it contains a contingent life event (i.e., an event that happened, but not necessarily one that had to happen, and one that turned life upside down), the story tells about life goals that come under pressure or presents new life goals, it describes at least two different perspectives on contingency, and it invites readers to reflect on or to question their own situation. In addition, we applied practical selection criteria: the story could be read in ten to fifteen minutes to engage patients less familiar with reading texts, and it presented various perspectives, for example regarding culture, religion, gender, and age of the protagonists. Using this method, we evaluated 42 texts and eventually identified 10 texts or fragments [25].

Three narratives are classically oriented (Greek) and/or play an important role in various religious traditions (Jewish, Christian, Islamic): “Orpheus,” “Job,” and “Yunus.” In the Greek story of Orpheus, the protagonist’s wife dies from a snake bite, after which Orpheus descends into the underworld to try to retrieve her [26]. Job, a righteous character, is successful in life, but in a short time, he loses all that is near and dear to him, and then rebels [27]. During a storm at sea, Yunus ends up in the belly of a large fish and fears death [28].

Four stories have a more contemporary style and are more accessible in a literary sense. In “The Fault in Our Stars” (John Green) [29], two people fall in love and enjoy life despite their incurable illness. The story “Code Catnip” (“Code Kattenkruid” in Dutch, by Jacques Vriens) [30] depicts a grandfather’s struggle to tell his grandchild that he will die. “The After Days” (“De nadagen” in Dutch, by A.N. Ryst) [31] describes the story of a son who witnesses the decline of his parents. In “Farewell from Phoebe” (“Afscheid van Phoebe” in Dutch, by Vonne van der Meer) [32] describes the difficult and lonely task of a woman who has to give birth to her dead child.

The three other stories are more fictional, appealing strongly to the reader’s imagination. In “Death of an old man” (Roald Dahl) [33], delusion and reality become intertwined when a pilot fights for his life after his plane is shot down. “The Metamorphosis” (“Die Verwandlung” in German, by Franz Kafka) [34] describes the thoughts of a man who wakes up one morning after being transformed into a huge insect. The story “The Ant’s Departure” (“Het vertrek van de mier” in Dutch, by Toon Tellegen) [35] describes how each animal in the forest personally deals with the sudden absence of the ant.

All ten stories were made available as a paper booklet. To support patients in their decision which story to read, a website was developed which included brief summaries and animated trailers of each story, developed by students at the University of the Arts, Utrecht. The website also contains the full written text of each story together with an audio version, produced by Thinium Audioboekproducties BV.

### Reading guide

As part of the intervention developed in the research project “In search of stories,” the patient can choose one of the selected literary texts and reflect on it at a meeting with a spiritual counsellor. To support this process, A.K., expert in the field of religious studies and cognitive narratology, developed a reading guide. The purpose of the reading guide is to enable patients to process the narrative and become acquainted with the diversity of views offered in the chosen story. At the same time, the reading guide also helps patients increase awareness of their own experiences and their identification with the subjects and situations in the narrative, thus stimulating the process of re-interpretation and meaning making. The reading guide contains three stages of reading and reflecting: close reading, recognizing, and connecting (Table 1).

**Table 1:** Narrative identification and reading guide

Narrative identification	Research area	Reading guide
Cognitive linguistic	Textual level of the narrative	Stage 1 Close reading
	Focus on linguistic perspective (Narrator-Characters)	“Who do you hear?”
	Focus on narrative world (Time-Space-Subjects-Situations)	“What do you see?”
	Focus on linguistic presentation Words-Concepts-Metaphors	“How is it presented in the text?”
Psychological	Interaction readers and texts	Stage 2 Recognizing
	Focus on subject identification	“In whom do I recognize myself?”
	Focus on situation identification	“Which event do I recognize?”
	Focus on detail/concept identification	“What moves me?”
Spiritual	Readers life narrative	Stage 3 Connecting
	Focus on reflection ((Emotional) response-thoughts-ideas) Focus on (re)interpretation (Concepts of meaning—Sources of meaning)	“How does the story affect you?”

#### Close reading

The close reading elements of the reading guide are based on previous experience by AK [36] in the context of reading spiritually oriented texts using a cognitive linguistic approach [37]. The focus of this stage is on the textual level of the narrative and the world of the story presented, stimulating patients to read consciously and carefully. In this textually oriented stage, patients are invited firstly to imagine the narrative world, and distinguish between “acts” that are recounted and “spoken text” that is given (“Who do you hear?”). In this way, their awareness is increased regarding the perspective of who “speaks” and through whose eyes we are looking. In doing so, the diversity of views is revealed as well as the choices made by the narrator and the characters [38]. Secondly, questions that focus on “time” and “space,” “subjects” and “situations,” as presented in the narrative, explicitly bring the narrative world to the fore (“What do you see”). The narrative world, or story-world, is the cognitive linguistic concept that refers to the complex mental representation readers construct during the process of reading [39]. Stage one concludes by focusing on the readers’ understanding of unfamiliar words and by looking at the various concepts, metaphors, and/or figures of speech (“How is it presented in the text?”).

#### Recognizing

Stage two focuses on the interaction between readers and texts and on their recognition of various aspects within the story. In this psychologically oriented stage, patients are invited to indicate which characters and situations they identify with, or which elements in the narrative emotionally move them. In order to stimulate patients’ involvement, the questions at this stage were formulated in terms of the first person. Empathizing with subjects (“In whom do I recognize myself?”), knowing similar events (“Which event do I recognize?”), or being touched by striking details (“What moves me?”) play a significant role in the process of meaning making. From a cognitive linguistic point of view, recognition is necessary to conceptualize and thereby to constitute meaning in order to form a coherent mental representation. From a psychological point of view, recognition is needed for the process of narrative identification.

#### Connecting

Stage three connects the narrative with the patient’s personal life story. In general, stories evoke all kinds of emotional responses and trigger all kinds of thoughts. Although not every narration moves or has a deep meaning for readers, it may evoke emotions and thoughts, support or question existing ideas, or open up new insights. At this spiritually oriented stage, the focus of the reading guide is on reflection and re-interpretation and therefore (as in stage one) is formulated in the second person (“How does the story affect you?”). Sub-questions focus on the elements in the story that touch the patients, emphasizing what this story personally means for their life narrative.

### The reading guide pilot study

In November and December 2019, we tested the reading guide presented above in a pilot study.

#### Ethical considerations

The pilot study’s research protocol was assessed by the Medical Ethics Review Committee of the Amsterdam University Medical Centers which decided that formal approval was not needed. Patients were orally informed about the study and by an information letter, including a warning of the potential risk of emotional stress and their right to withdraw from the study at any time. Patient confidentially was ensured and all collected data were coded and stored in a protected database. Written informed consent was obtained from each participating patient.

#### Procedure

Patients were recruited from the Department of Medical Oncology of the Amsterdam University Medical Centers, Location AMC, The Netherlands. We approached patients who were diagnosed with advanced, incurable cancer, and receiving palliative treatment and/or best supportive care. Based on their willingness, eight patients initially agreed to participate in this study. Due to personal circumstances, three patients, of which two female patients, refrained from participation, resulting in a purposive sample of five male patients. The first contact with patients was established by the outpatient unit manager of the Department of Medical Oncology during a visit in the context of their treatment. After gaining initial consent, each patient was contacted by phone by the project researcher (AK), who provided further information on the study and made a personal appointment for an interview if patients agreed. All consenting patients were offered the opportunity to be interviewed either at the hospital or at home.

The session consisted of the patient choosing and reading one of the presented stories, discussing the narrative using the reading guide, and concluding with debriefing questions to evaluate the session. All sessions were audio recorded. Since neither the supportive website nor the booklet was yet available, patients were offered a random choice of three stories from the selection of ten: one of the three classically oriented, one of the four contemporary texts, and one of the three more fictional stories. At the request of one patient, all ten stories were shortly introduced at that particular meeting.

The interviews were conducted according to a predetermined protocol in which the reading guide questions functioned as interview questions. The session started with getting acquainted, followed by introducing the study, choosing, and reading one of the three stories by the patient, stage one “close reading,” stage two “recognizing,” stage three “connecting,” with a final evaluation and conclusion.

#### Data analysis

The audio recording of each session was thematically analyzed regarding its conceptual content by means of a template analysis, as described by, among others, King [40]. This type of thematic analysis is very suitable for qualitative research in the setting of patient-centered medical care, as also shown by studies of, among others, Brooks et al. [41] in the context of qualitative psychology, or De Vries et al. [42] in the context of patients with advanced cancer. We used Atlas.ti-software to encode and analyze the data of the recorded conversations [43].

Template analysis involves the application of a coding “template” with which qualitative data, such as interview transcripts, are thematically coded and interpreted. The “template” itself consists of a number of themes identified by the researcher. The analysis often starts with some “a priori” themes, which are expected to be relevant for the analysis. After reading and rereading the data, these (deductive) themes can be revised and refined, and supplemented with inductive themes which emerge in the transcripts. Ordering and specifying themes and sub-themes creates a hierarchically structured template which is applied to all obtained data (i.e., the interviews).

The first two interviews were coded by AK based on the questions of the reading guide. This initial template was discussed with MSR, after which five “a priori” (deductive) main themes and five sub-themes (of the first two main themes) were established: (1) story choice, (2) narrative identification, (3) experience of contingency, (4) concepts of meaning, and (5) sources of meaning [44]. The sub-themes of story choice were the specific chosen stories, and the sub-themes of narrative identification were the identification with subjects and/or situations in the story (Table 2).

**Table 2:** Template analysis

Main themes	Sub-themes
1. Choice of story	1.1 Job
	1.2 The Fault in Our Stars
	1.3 Code Catnip
	1.4 Death of an old man
2. Narrative identification	2.1 Subject
	2.2 Situation
3. Experience of contingency	3.1 Loss of life goals
	3.2 Impending death
	3.3 Uncertainty
4. Concepts of meaning	4.1 Fate
	4.2 Life goals
	4.3 Quality of life
	4.4 Death
5. Sources of meaning	5.1 Christian worldview
	5.2 Buddhist worldview
	5.3 Secular worldview

Based on a detailed reading of all five interviews by AK, the template was further discussed and refined with help from MSR, after which ten inductive sub-themes were added to the final template for three of the main themes (Table 2): sub-themes loss of life goals, impending death, uncertainty (aspects of experience of contingency); sub-themes fate, life goals, quality of life, death (specific concepts of meaning that came to the fore); sub-themes Christian worldview, Buddhist worldview, Secular worldview (specific sources of meaning which were mentioned). The analysis and interpretation of the data by AK were discussed with EvW, MSR, and HvL.

The main themes of the template match the research concepts described in the “Introduction” section. The specific choice of one of the selected stories and the interaction with the text can provide insights into the process of narrative identification. The specific content of the experience of contingency and the way in which the experience was discussed can provide insights into the way in which the patient connects the experience of contingency to concepts of meaning, and, in a larger context, to his or her worldview.

## Results

### Patients

Five patients, all male and aged between 54 and 74, agreed to take part in the pilot study. Three had a Christian upbringing, but were not religiously active; the other two had no specific religious background. The interviews took place at each patient’s home according to their preference, the main reason being that they perceived their home as more suitable for discussing personal and inner life. Each interview lasted 75–90 min in total, of which 60 min were spent reading and discussing the story. Most patients were tired after 60 min because of the intensity of the conversation. We note that future meetings of this intervention part will have to be more efficient. Given the availability of the website and the booklet, less time will be needed for the project’s introduction, and patients can choose and read a story prior to the meeting.

### Choice of story

All five participants chose a story that was close to their personal experiences. For example, the choice for the story of “Job” was prompted by the patient’s past religious education, combined with a strong identification with the main character: “Job experiences “It befalls me”. Because that is how you experience it with cancer.” (P1, aged 65).

Two patients chose “The Fault in Our Stars,” a story about an impossible love affair. For one patient (P2, aged 74), the memory of his love for his deceased wife and the impossible love for someone else in the present established a strong association with the chosen story. The other patient (P3, aged 54) was interested in all ten stories. The love for his wife, who he had lost to cancer, appealed directly to the story of “Orpheus.” However, the combination of a new love and his own illness made “The Fault in Our Stars” more appropriate for him. The “Code Catnip” story was chosen by another patient (P4, aged 72) based on an experienced dilemma in the present and on his recognition of the main character. Like the acting subject in the narrative, he is a grandfather who finds it difficult to explain to his grandchild that he is terminally ill. The patient who chose the story “Death of an old man” (P5, aged 70) had a strong preference for narratives situated in the Second World War.

During all five interviews, the first stage of the reading guide was of limited value. The questions “Who do you hear?,” “What do you see?,” and “How is it presented in the text?” functioned mainly as a means of checking whether the story had been properly understood. Since the narrative had just been introduced and had only just been read, there was hardly any need to explicitly re-activate the story-world. Nevertheless, during the meeting, parts of the story were regularly referred to, thus bringing the mental representation of the narrative world (back) to the forefront of the conversation. In particular, the question about subjects, situations, and events (“What do you see?”) acted as a point of reference during the sessions, maintaining the focus on the theme of experiences of contingency, especially at times when the conversation deviated too much.

### Narrative identification and experiences of contingency

In response to the second stage questions “In whom do I recognize myself?,” “Which event do I recognize?,” and “What moves me?,” almost all participants answered immediately, often intuitively. For example, in the story of “Job,” known passages were quoted almost immediately by the patient, such as “Naked I came from my mother’s womb, and naked shall I return there” (Job 1:21). For this patient, the quotation meant the things that are fixed in life and cannot be changed. Highly emotionally charged statements also emerged: “Nobody asks to become ill, it happens to you and Our Lord did not predetermine it.” (P1, aged 65). This patient recognized the life experiences of the character of Job but not Job’s anger or revolt. The patient also recognized the experience of different perspectives (in the story these are articulated by the locations in heaven and on earth), but he did not evaluate his personal situation as being “unfair.” His recognition of himself in the narrative subject Job and his empathy with the situation of the main character quickly led to personal experiences of his illness. For example, the parallel to the friends of Job, who “interrogate” Job about his beliefs, evoked the experience of similar reactions of others in the patient’s personal environment: “That’s how it happens in real life. Opinions [on how to deal with your illness] come from all sides. But what do you do with them [opinions of others]?” (P1, aged 65).

Other salient examples of textual recognition and personal identification occurred after reading “The Fault in Our Stars”; the narrative evoked strong emotions in both patients. For one, the story initially appealed to the concept of love and sexuality. He pointed out that being seriously ill does not mean that people can no longer have sex, a theme also reflected in the story. Moreover, it turned out that he strongly identified with the narrative protagonist Augustus, not so much because of appearances or specific character traits, but mainly because of Augustus’ views on life: “It’s about living in the here and now, getting cancer is just bad luck.” Although the story initially evoked romantic connotations and was reminiscent of his love in the here and now, during the conversation, his memories of his first wife and of his life as a whole predominated: “I no longer have a future, only a past.” This patient still thought his life was worth living, although as in the story, the practical limitations of, for example, a stoma did not make it any easier: “I just expect to muddle through until it’s over.” (P2, aged 74).

For the other patient, the narrative subject itself was not the point of recognition, but a detail in the story: “I got up, dragging my body and the cart across the carpet that was older than Augustus would ever be, and I knelt at the base of the chair and put my head in his lap and hugged him by the waist” (The Fault in Our Stars, chapter 13). The recognition of the thought about the carpet, and thereby the finiteness of human existence, caused this patient to think about the duality that illness entails. On the one hand, there are feelings of lust for life, cheerfulness, love, and also sexuality: “It also ends nicely in the story, when they say, “Let’s try to make love”; “try nothing,” I said, “just do it.”” (P3, aged 54). On the other hand, there is the continued presence of impending death and its consequences for his family and loved ones: “The realization (of death) hurts because I project that realization onto someone else ...” The fact that he was now confronted with cancer for the second time in his life, but this time himself, resulted in the prominence of the subject of his own mortality during the conversation: “When my wife passed away I really thought, well this was it. I considered myself almost immortal, this is not going to happen to me. That is of course not based on anything, but we have had our share.” (P3, aged 54). In retrospect, the patient’s identification with an apparently insignificant detail related to a textual comment about the age of carpet activated all kinds of affectionate emotions and experiences of contingency in his life story.

For the patient who chose the story “Code Catnip,” the identification process ran parallel to his reasons for choosing this story. Like the main character, he is a grandfather who has a hard time communicating with his (grand)children about his illness and inevitable death: “Well, of course I see the game playing between an elderly man who is ill and probably also has a chance of dying, who also loses his quality of life, but yet still empathizes with the life of this child, and that is also what you have to do…” Besides communicating about his health and prospects, this patient found it difficult to deal with the day-to-day consequences of his illness: “But what bothers me the most is my loss of quality of life. I didn’t have to work that hard anymore and at the age of 72 I had a pretty good life. In fact, it all went well. I travelled all over the world, everything went fine. That was suddenly taken away. And that bothers me.” His deteriorating quality of life had made him decide to end his life through euthanasia when the time is right, just like the main character in the story: “I’m aware that I have my own choice too ... And if it has to happen, it will happen. And I am confident that I will be helped if necessary.” (P4, aged 72).

For this patient, the strength of the textual identification lay in the conceivability of the story as a whole. He understood the protagonist’s struggle with himself, with his environment, with his own death, and he recognized his own final choices.

When discussing the story “Death of an old man,” the identification process was mainly through recognizing the actions and situations in which the main character found himself. The chosen story, in which delusion and reality intertwine, details the dire situation of the protagonist who hangs from a parachute after his plane has been shot down. While he fights for his life, he ends up in a kind of mud pool. The patient in question linked the narrative’s evoked images metaphorically to his own situation. He had been diagnosed with cancer for the fourth time in his life and, a few years earlier, had also suffered a brain hemorrhage. He associated the shot pilot in the story with the situation that had befallen him; the pilot’s hanging from a parachute he associated with his own helplessness, the pool of water and mud in the story with his own struggle: “That you have the strength to escape, and come out of the pool.” When discussing the end of his own life, and a possible afterlife, this patient repeated an image from the story, as a kind of visionary future reality in which he hopes to meet his wife again: “There is always someone waiting for me … that must be (in the story) a woman waiting for him.” (P5, aged 70).

### Concepts of meaning and worldview

In the third stage of the reading guide, patients were asked the question: “What does the story mean to you?” This question is intended to stimulate the connection of the evoked experiences with the reader’s own ideas and insights, and also as a first step towards reflection and reinterpretation. As a separate question, it turned out to be difficult for the patients to answer. However, it did evoke reactions about the perception of contingency on the one hand and the patient’s view of life on the other. In a sense, it was striking that no matter how different each person’s life was and how distinct each personal situation was regarding the illness, both the contingent nature of the experience and the view of life were formulated in similar terms.

The experience of contingency in relation to incurable cancer was, more or less, called “fatum,” the fate that befalls you without anyone being guilty: “it happens to you ... it is my fate and you have to make do with that” (P1, aged 65), “it is bad luck ... just by chance” (P2, aged 74), “coincidence, this just happens, there is no reason for it, just like that ... that will not happen to me” (P3, aged 54), “pure bad luck” (P4, aged 72), “it happens to you” (P5, aged 70).

When talking about these initial reactions, about what being seriously ill does to you in an emotional or spiritual sense, a more differentiated picture emerged than a resigned determination of fate alone. One patient mentioned grief: “Then your world collapses. I couldn’t read anymore, I couldn’t remember any more books ... that moment, from my first hearing the death sentence” (P1, aged 65); panic: “In the beginning it was blind panic. Not really blind panic, but when I was diagnosed and discovered it was not really curable, then I experienced it as something really serious, just because my wife died” (P3, aged 54); incomprehension: “I’ve always said: what have I done wrong? But yes, so be it” (P5, aged 70); and anger: “Setbacks, with which you have learned to deal with, you can accept. But this is too much. This is too much, this is the straw that broke the camel’s back” (P4, aged 72).

The description of the emotion associated with the experience of contingency for patients also provided a brief insight into their worldview. Such as the thought “what have I done wrong,” which turned out to be mainly about this patient’s struggle with his religious views. Although he had not believed in a god for a long time, his incomprehension turned out to be religiously formulated: “No, I sometimes think, why is he (God) punishing me for the fourth time?” (P5, aged 70). Another patient, who indicated that he was no longer religious, at least not actively, verbalized a clear spiritual attitude to life in the course of the conversation, in which his Christian worldview resonated: “I do not look at life negatively. I don’t look at it as finite either. I know, I’ve never blamed (God) ... I still think there’s something more.” (P1, aged 65). Although he had a Christian youth, and although the Buddhistic worldview appealed to him, another patient placed his situation in a secular perspective: “I expect I expect to keep struggling until the end.” (P2, aged 74).

The patients without a religious background referred to their worldview in a secular way, pointing to the existential wellbeing in the here and now and secular views on afterlife: “I don’t believe in an afterlife, and I don’t believe in a god. No, I believe it should happen here and now.” (P3, aged 54); “There is no answer to anything, no, you have to find that yourself … after this life it is over” (P4, aged 72).

## Discussion

We report our first experiences with reading and discussing exemplary literary texts that contain experiences of contingency with advanced cancer patients, using a newly developed reading guide to assist the process of narrative identification.

All five patients showed some form of recognition in reaction to the chosen text, which ranged from textual recognition of situations in which the characters find themselves, to specific thoughts and ways of thinking, from small details in the narrative world to the story as a whole. Such personal connections of readers with the stories they have read are studied extensively in the research on literature and narrative discourse, witness the aforementioned studies on literary response by Oatley [19], on audience identification by Cohen [19], or identification with characters by Van Krieken et al. [38]. Some authors, like Frank [45], more specifically emphasize how stories invite readers to adopt different perspectives and can be used to enhance human lives and relations. The same author emphasized in his former research the importance of the personal stories told by people afflicted with illness and its therapeutic effect on ill people’s lives [46]. However, the intervention tested in this study above all aimed to activate patients own experiences of contingency, by means of reading and discussing one of the selected stories. For example, the story of Job triggered an identification with a similar situation of inexplicable suffering. “The Fault in Our Stars” evoked empathy with the protagonist’s ideas, appealing to the patient’s own thoughts about love and life. The same story touched another patient with a striking detail, which opened up various thoughts on his view of life. “Code Catnip” appealed to a personal recognizable reality because of the story as a whole, instead of one single detail. “Death of an old man” stimulated a metaphorical identification with the narrated situation, the patient’s actual life being a struggle.

The combination of narrative recognition and identification with highly emotional reactions, after reading similar stories by patients with an incurable disease, shows great similarities with the observations made by Gerald West during sessions with discussion groups of HIV-infected patients in South Africa [47]. In one of the group meetings, after reading and exploring chapter three of the book of “Job,” a participant said, “We are like Job; we are good people who were not looking for this thing, and yet now we are infected” (West, 2015, pp. 63) [48]. Just as in our patient who chose the story of Job, this participant strongly identifies with the suffering of the protagonist Job, and at the same time stated that his own life differed. And just like our patient, who mentioned the many comments from his personal environment on dealing with his illness, after reading the textual passages of Job and his friends participants of the discussion group in South Africa elaborately detailed their experiences of being judged by their families and friends and churches (West, 2015, pp. 64). The examples show that the story of Job seems to evoke similar responses of recognition by different readers, detailing experiences of contingency in view of a severe disease.

The results of the pilot study show that narrative identification on a psychological and spiritual level may also occur. The various emotions regarding their illness (such as grief, panic, incomprehension, anger), the diverse concepts of meaning (such as fate, life goals, quality of life, death), and connections to their worldview, as expressed when discussing the text, bear witness to this. The emotions, concepts of meaning, and sources of meaning correspond to the three domains of meaning making, mentioned by La Cour and Hvidt [49]. They distinguish a secular, religious, and a spiritual domain that partially overlap each other. Calling something “fate,” for example, is not exclusive to a religious worldview, nor is the concept “quality of life” exclusively secular. More generally speaking, the words belong to the vocabulary of meaning making. The meaning of these concepts depends on the specific situation of each individual patient and relates to their personal worldview.

In other words, the textual narrative world of each story provided words and images which enabled the patients individually to express their own experience of contingency and to rethink what they had endured. In this way, the interaction between literary texts and the story of patients stimulates the reflection on their own experiences, enabling them to place these experiences in a larger context of meaning making and view of life [50].

The reading guide, as a reviewing tool and conversation template, demonstrated its potential to activate the recognition of experiences of contingency. The conceptualization of the narrative reality (stage 1 of the reading guide) and the direct questioning of recognition and identification (stage 2 of the reading guide) give focus and direction to the conversation. Especially the strong and emotional responses of patients to the reading guide questions “In whom do I recognize myself?,” “Which event do I recognize?,” and “What moves me?” seem to open up a powerful connection to personal experiences of contingency and view of life. Together with the question about the meaning of the story for the reader (stage 3 of the reading guide), thoughts about meaning making and the patient’s worldview are activated in a purposeful way.

Although the implementation of stage one, close reading, may at first sight seem limited because the story had just been read by the patient, in a setting where the story is read at an earlier moment, this stage of the reading guide will probably contribute more when reactivating the story.

Moreover, it can be argued that starting with the specific details of the story itself—rather than directly with the patient story—facilitates patients when telling their story; one of the participants explicitly mentioned that he was pleased to start the conversation with the written story rather than with his own experiences. Someone else’s (literary) story gave him the opportunity first to distance himself from his own story and perspective, and secondly, to look at his own situation through new eyes [51].

In stage 3, the question “what does this story do to you” turned out to be difficult for patients to answer directly. Patients were unable to easily place their experiences in a larger context of meaning making and worldview. However, during the interviews, when further discussing the story in relation to their own experiences, various concepts of meaning were explicitly mentioned, sometimes directly related to a worldview (e.g., Christian worldview, Buddhist worldview, or Secular worldview). Although our study used a different conversation template, our findings are consistent with the study by La Cour and Schnell [44]. Their empirical research show that it is helpful during a semi-structured interview to specifically question participants on their sources of meaning, since it appears to be difficult for participants to express this on their own.

Several limitations of our study should be acknowledged. Our sample consisted of five Dutch men, whereas a larger group of patients, with diverse cultural backgrounds, and a more balanced composition regarding gender could have widened the results of the thematic analysis. It should also be noted that with the exception of the stories “Farewell from Phoebe” and “The Ant’s Departure,” the literary stories were mainly from a male perspective. Patients who are not male might respond differently to the male-voiced texts, and vice versa. In the future, additional texts that reflect perspectives besides the male perspective might also need to be included. Due to the small sample of participants, we did not reach data saturation in the thematic analysis. However, rather than aiming for data saturation, the thematic analysis was employed to explore if and how patients relate to the stories they were presented with. Future research should investigate the full breadth and depth of the themes that are evoked by these stories.

## Conclusion

In conclusion, first experiences with our newly developed reading guide designed to support a structured reading of stories containing experiences of contingency suggest that it may help patients to express their own experiences of contingency and to reflect on these experiences. Further research is needed to investigate whether introducing recognizable narratives that represent experiences of contingency and discussing a selected story with a spiritual counsellor contribute to improving the supportive care and quality of life of advanced cancer patients.

## Data Availability

Not applicable
